# Physicochemical Characterization and Evaluation of Gastrointestinal In Vitro Behavior of Alginate-Based Microbeads with Encapsulated Grape Pomace Extracts

**DOI:** 10.3390/pharmaceutics15030980

**Published:** 2023-03-18

**Authors:** Josipa Martinović, Jasmina Lukinac, Marko Jukić, Rita Ambrus, Mirela Planinić, Gordana Šelo, Ana-Marija Klarić, Gabriela Perković, Ana Bucić-Kojić

**Affiliations:** 1 Faculty of Food Technology Osijek, Josip Juraj Strossmayer University of Osijek, F. Kuhača 18, HR-31 000 Osijek, Croatia; 2 Faculty of Pharmacy, Institute of Pharmaceutical Technology and Regulatory Affairs, University of Szeged, H-6720 Szeged, Hungary

**Keywords:** encapsulation, ionic gelation, particle characterization, winery residues, phenolic compounds, simulated digestion in vitro

## Abstract

Grape pomace is a byproduct of wineries and a rich source of phenolic compounds that can exert multiple pharmacological effects when consumed and enter the intestine where they can then be absorbed. Phenolic compounds are susceptible to degradation and interaction with other food constituents during digestion, and encapsulation may be a useful technique for protecting phenolic bioactivity and controlling its release. Therefore, the behavior of phenolic-rich grape pomace extracts encapsulated by the ionic gelation method, using a natural coating (sodium alginate, gum arabic, gelatin, and chitosan), was observed during simulated digestion in vitro. The best encapsulation efficiency (69.27%) was obtained with alginate hydrogels. The physicochemical properties of the microbeads were influenced by the coatings used. Scanning electron microscopy showed that drying had the least effect on the surface area of the chitosan-coated microbeads. A structural analysis showed that the structure of the extract changed from crystalline to amorphous after encapsulation. The phenolic compounds were released from the microbeads by Fickian diffusion, which is best described by the Korsmeyer–Peppas model among the four models tested. The obtained results can be used as a predictive tool for the preparation of microbeads containing natural bioactive compounds that could be useful for the development of food supplements.

## 1. Introduction

Grapes are among the richest and most diverse sources of phenolic compounds in the plant kingdom, which can be used in various foods, cosmetics, and pharmaceutical products [1,2,3]. The processing of grapes into wine or juices generates a large number of by-products, such as grape pomace (GP), which is a potential feedstock for biorefineries for the sustainable production of biofuels and biobased products (chemicals, materials, biopolymers, food, feed, pharmaceuticals, nutraceuticals) due to its chemical composition (proteins, fats, sugars, fibers, polyphenols, minerals, vitamins, fatty acids, etc.) [4]. The effective utilization of food industry residues, including GP to recover phenolic compounds and produce various other value-added products, is increasingly being considered a sustainable waste management strategy. The valorization of by-products is of great interest for tackling the problems arising from the disposal of GP and for achieving the goals of the circular bioeconomy [1,5,6]. Phenolic compounds found in GP can be divided into: flavonoids (flavanols, flavonols, anthocyanins, isoflavones, etc.), phenolic acids (hydroxycinnamic and hydroxybenzoic acid derivatives), and stilbenes (mainly resveratrol) [7,8,9]. These compounds have many beneficial properties on human health, such as antioxidant [10], antitumor [11], and anti-inflammatory [12] properties, as well as low bioavailability [13]. During digestion, phenolic compounds undergo various changes due to conditions in the mouth, stomach, and intestines, such as the transformation of their chemical structure, which affects their bioaccessibility and bioactivity [3]. To ensure their stability and controlled release in the digestive system so that they can exert their beneficial properties [14,15], phenolic compounds can be encapsulated [3,16].

Encapsulation is a process in which an active ingredient is coated or crosslinked with a polymer (coating) that can preserve that active ingredient [17]. The coating used must be suitable not only for the encapsulated compound, but also for the purpose for which the encapsulation is used [16,18]. There are many coatings that can be used, such as proteins, lipids, and polysaccharides. However, today, there is a great tendency to use coatings of natural origin instead of synthetic ones for encapsulation, so the coatings are often isolated from natural sources [3]. The polysaccharides alginate, gum arabic, chitosan, and protein gelatin are coatings that fit this profile. Alginate is a linear polymer composed of β-D-mannuronic acid (M blocks) and α-L-guluronic acid (G blocks) linked by β-(1-4)-glycosidic bonds [19]. It is mainly found in the cell walls of brown algae and is usually extracted and processed in the form of a sodium salt [20]. The composition and method of distribution of the M- and G-blocks are responsible for its physicochemical properties, such as viscosity, water absorption capacity, and salt-gel transition, which enable its wide use in the cosmetic, pharmaceutical, food, and agricultural industries [19,20]. Gum arabic (GA) is a natural, branched-chain heteropolysaccharide gum exudate from acacia trees. The backbone of gum arabic consists of 1,3-linked β-D-galactopyranosyl units and, connected to the main chain via 1,6-links, side chains consisting of two to five 1,3-linked β-D-galactopyranosyl units [17]. Due to its highly branched structure and covalent linkage to protein structures, gum arabic is used as an emulsifier and stabilizer in the food and pharmaceutical industries, as well as a food additive, drug-delivery agent, biomaterial in tissue engineering, and more [21,22]. Chitosan (CHIT) is a linear polysaccharide composed of D-glucosamine monomers with randomly arranged β-(1 → 4)-linked N-acetylglucosamine substituents. This polysaccharide is particularly compliant to structural modification to yield materials of different mechanical and physical properties [20]. Due to its easy modification, biodegradability, negligible toxicity, and mucoadhesive property, chitosan is widely used by the pharmaceutical industry [19,20]. Gelatin (GEL) is obtained by the partial hydrolysis of collagen from many animal sources of gelatin such as cattle, pigs, and fish. Among them, fish gelatin is one of the most ideal materials for the production of hydrogels used in the food industry. Moreover, it can be obtained from by-products of the fishing industry (heads, guts, skin, and bones), which can contribute to waste reduction [20,23,24].

The coatings used in encapsulation affect many properties of the particles produced, such as geometrical parameters, texture, and surface morphology, and these properties also play a role in the in vitro release behavior of the encapsulated compounds [25,26]. The release of encapsulated bioactive compounds from prepared particles should be controlled, i.e., the release should occur at the target site where the biological properties of the compounds can be achieved [17]. Knowledge of the release kinetics is important for bioactive compound delivery systems, and various mathematical models have been developed to describe and simulate the release process of bioactive compounds in drug delivery systems or food delivery systems [27]. Four main mechanisms may play a role in the release of bioactive compounds from encapsulated particles, including dissolution, diffusion, swelling, and erosion [28]. Furthermore, mathematical modeling plays an important role in predicting the release rate and release profile of bioactive compounds while reducing the number of experiments required [27].

In this work, a phenolic compound-rich grape pomace extract (GPE) was encapsulated with different alginate-based coatings by ionic gelation. The objective of the work was to determine how the encapsulated phenolic compounds incorporated into the GPE behaved in an in vitro digestion simulation without enzymes during the oral, gastric, and intestinal phases. The influence of physicochemical properties, such as the size and shape of microbeads, texture, morphology and structure on in vitro release, was also investigated. Finally, the release kinetics of the phenolic compounds were described by appropriate mathematical models based on Fick’s diffusion law. To the best of our knowledge, there is little literature on the encapsulation of phenolic-rich grape pomace extracts by ionic gelation, while there are data on the encapsulation of phenolic compounds from grape pomace or its components (skins and seeds) by other encapsulation techniques (spray-drying and freeze-drying), which are more expensive than the method studied in this article. Therefore, the contribution of this work is the use of waste streams from wineries for the production of high-quality phenol-rich encapsulated particles using an economically and energetically efficient encapsulation method such as ionic gelation, and a comprehensive study of the behavior of the encapsulated phenolic compounds during in vitro digestion.

## 2. Materials and Methods

### 2.1. Chemicals and Reagents

Alginic acid sodium salt from brown algae (low viscosity); gum arabic (powder); gelatin from cold water fish skin; medium-molecular-weight chitosan (deacetylated chitin); gallic acid monohydrate (98+% A. C. S. reagent); n-octanol; sodium sulfite anhydrous (98–100%, p.a.); DL-arabinose; 2,2′-azino-bis(3-ethylbenzothiazoline-6-sulfonic acid) diammonium salt (ABTS); 2,2-diphenyl-1-picrylhydrazyl (DPPH); standards for UHPLC analysis (-)-epicatechin; epicatechin gallate; gallocatechin gallate; (+)-catechin hydrate; phenolic acids (gallic, syringic, ellagic, *p*-coumaric, *o*-coumaric, caffeic, ferulic and *p*-hydroxybenzoic); resveratrol, rutin hydrate; and kaempferol were purchased from Sigma Aldrich (Saint Louis, MO, USA). Folin and Ciocalteu’s phenol reagent was purchased from CPA chem (Bogomilovo, Bulgaria); 96% ethanol (p.a.) from Lab Expert (Shenzhen, Guangdong, China); sodium hydroxide (p.a.) from Kefo (Sisak, Croatia); and sodium hydrate carbonate (p.a.) and iron(II)sulfate heptahydrate from Kemika (Zagreb, Croatia). Sodium carbonate anhydrous (p.a.); potassium chloride (p.a.); potassium dihydrate phosphate (p.a.); sodium chloride (p.a.); magnesium chloride 6-hydrate (p.a.); calcium chloride 2-hydrate (p.a.); calcium chloride anhydrous (p.a.); sodium acetate anhydrous (p.a.); sodium nitrite; sodium hydroxide; D(+)-glucose; and acetone were obtained from Gram Mol (Zagreb, Croatia). Glacial acetic acid (99.5%) was purchased from Macron Fine Chemicals (Gliwice, Poland); trisodium citrate dihydrate, ethylenediaminetetraacetic acid iron(III) sodium salt, and sulfuric acid (96%) from T.T.T. (Sveta Nedelja, Croatia); ammonium carbonate, vanillic and 3,4-dihydroxybenzoic acids, and quercetin, as well as sodium tetraborate decahydrate (99.5%, for analysis), Trolox, D(+)-sucrose, D(-)-fructose, hexadecyltimethylammonium bromide (99+%), and iron(III)chloride hexahydrate (99+%, for analysis) from Acros Organics (Geel, Belgium); and hydrochloric acid (37%) and n-hexane from Carlo Erba Reagents GmbH (Emmendingen, Germany). HPLC grade acetonitrile and 2-ethoxyethanol were from Fisher Chemical (Loughborough, UK) and ultra-gradient grade methanol from J.T. Baker (Arnhem, The Netherlands). Sodium laurylsulfate was obtained from Carlo Erba Reagents S.A.S. (France); sodium phosphate dibasic anhydrous, 98+% extra pure from Thermo Fisher Scientific (Fair Lawn, New Jersey, USA); procyanidins B1 and B2, oenin chloride, myrtillin chloride, kuromanin chloride, petunidin chloride, callistephin chloride, and peonidin-3-*O*-glucoside chloride from Extrasynthese (Genay, France); 2,4,6-tris(2-pyridyl)-s-triazine (TPTZ) for spectrophotometric det. of Fe (≥98.0%) from Sigma Aldrich (Switzerland); ammonium persulfate from Honeywell (Seelze, Germany); aluminum chloride hexahydrate from Alfa Aesar GmbH & Co KG (Kandel, Germany); and 1-butanol from Fisher Scientific (Loughborough, UK).

### 2.2. Grape Pomace Sample

GP are residues left behind when grapes are processed into wine and consist of pulp, seeds, skins, and sometimes stems. GP of the Cabernet Sauvignon variety was collected from a local winery (Erdut Winery, Erdut, Croatia, 2018 harvest). After collection, the GP was air-dried and stored at room temperature. Before use for the experiments, it was ground to a particle size of ≤ 1 mm using an ultracentrifugal mill (Retsch ZM200, Haan, Germany).

### 2.3. Chemical Composition of Grape Pomace

The chemical composition of GP was studied, and the analyses were performed in triplicate, with the results expressed to the dry weight of the sample to provide a more accurate representation. The dried and grounded GP was used directly to determine the dried matter content (91.91 ± 0.01%); ash content [29]; content of neutral detergent fibers (NDF), acid detergent fibers (ADF) and acid detergent lignin (ADL) [30]; crude proteins content [31]; total nitrogen (TN) and organic carbon (TOC) content; and free fats content [32]. Modifications of the original methods and a detailed procedure for the above analyzes were presented in the article published by Šelo et al. [6]. The results of the chemical composition were expressed as the mean of the replicates ± standard deviation (SD).

#### 2.3.1. Phenol-Rich Grape Pomace Extract Preparation

The extraction of phenolic compounds from GP was performed in a shaking water bath (Julabo, SW-23, Germany) at 80 °C and 200 rpm for 2 h. A 50% aqueous ethanol solution with a GP–solvent ratio of 40 mL/g was used as solvent. After extraction, the samples were centrifuged at 11,000× *g* for 10 min (Z 326 K, Hermle Labortechnik GmbH, Germany). The supernatant obtained (liquid GPE) was then used to determine the content of the total and individual phenolic compounds, total flavonoids (TF), and total extractable proanthocyanidins (TPA), and to determine the antioxidant activity (AA) of the GPE. Anthocyanin profile analyses were also performed in liquid GPE prepared by the same procedure, but distilled water was used as solvent instead of 50% ethanol. All extractions were performed in three replicates.

#### 2.3.2. Determination of Total Phenolic Content (TPC)

TPC was determined using the Folin–Ciocalteu colorimetric method, according to the microscale protocol described by Waterhouse [33], with some modifications. Briefly, 40 µL of the extract was mixed with 3160 µL of water and 200 µL of Folin–Ciocalteu reagent. After 8 min, 600 µL of 20% (*w/v*) Na_2_CO_3_ was added, and the mixture was incubated at 40 °C for 30 min. Absorbance was measured at 765 nm against a blank containing water instead of the sample. The final results were expressed as gallic acid equivalent per dry basis of GP (mg_GAE_/g_db_).

#### 2.3.3. Determination of Total Flavonoids Content (TFC)

TFC was determined by the spectrophotometric method with aluminum chloride, according to Marinova et al. [34], with minor modifications. A volume of 0.5 mL of GPE was mixed with 2 mL of water. Then, 0.15 mL of a 5% (*w/v*) sodium nitrite solution was added, and after 5 min, 0.15 mL of 10% (*w/v*) aluminum chloride solution as well. After exactly 6 min, 1 mL of 1 M NaOH was added, and the mixture was diluted with 1.2 mL of distilled water. The absorbance was measured at 510 nm against a blank. The final results were expressed as (+)-catechin equivalent per dry basis of GP (mg_CE_/g_db_).

#### 2.3.4. Determination of Total Extractable Proanthocyanidins (TPA)

Based on the acid–butanol reaction with GPE, TPAs were determined according to the method published by Škerget et al. [35], with minor modifications. Briefly, 0.5 mL of the GPE was added to 5 mL of an iron(II)sulphate heptahydrate solution prepared by dissolving 77 mg of FeSO_4_(H_2_O)_7_ in 500 mL of HCl-butanol solution (2:3, *v/v*). The mixture was stirred and incubated in a water bath at 95 °C. After 15 min, the mixture was cooled under water and the absorbance was measured at 540 nm against a blank sample containing distilled water instead of the sample. The TPA content was calculated according to the molar extinction coefficient and the molar weight of cyanidin, and the final results were expressed per dry basis of GP (mg/g_db_).

#### 2.3.5. Determination of Individual Phenolic Compounds and Anthocyanins

Ultra-high performance liquid chromatography (UHPLC Nexera XR, Shimadzu, Japan) using a photodiode detector (PDA) was used for the qualitative and quantitative analysis of individual phenolic compounds and anthocyanins in GPE. Separation was performed using a reversed-phase Kinetex^®^ C18 core-shell column (100 × 4.6 mm, 2.6 µm, Phenomenex, Torrance, CA, USA). Samples were filtered through 0.45 µm membranes before UHPLC analyses (Chromafil Xtra PTFE, Macherey-Nagel GmbH & Co. KG, Dueren, Germany). Data were processed using LabSolutions 5.87 software. The individual phenolic compounds and anthocyanins were identified by comparing their retention times and UV-Vis spectra with those of authentic standards analyzed under the same chromatographic conditions. Quantification was performed using the calibration curves prepared with the external standards. Hydroxycinnamic acids were determined at 276–277 nm, hydroxybenzoic acids at 252–280 nm, flavonols at 365–370 nm, flavan-3-ols at 273–277 nm, procyanidins at 278 nm, and stilbenes at 305–323 nm. Anthocyanins, callistephin chloride, kuromanin chloride, peonidin-3-*O*-glucoside chloride, myrtillin chloride, oenin chloride, and petunidin chloride were determined at 503, 513, 517, 523, 526, and 531 nm, respectively.

Individual phenolic compounds were determined by following the previously published protocol by Bucić-Kojić et al. [36]. Separation was performed with a linear gradient of two solvents: Solvent A (50% methanol/50% acetonitrile, *v/v*) and Solvent B (1.0% acetic acid in water, *v/v*). A linear gradient was performed at 30 °C with a flow rate of 1 mL/min from 5% to 30% B in 25 min, from 30% to 40% B in 10 min, from 40% to 48% B in 5 min, from 48% to 70% B in 10 min, from 70% to 100% B in 5 min, isocratic at 100% B for 5 min, followed by a return to baseline conditions (10 min) and column equilibration (12 min). The injection volume was 20 µL.

The determination of anthocyanins by the UHPLC method was performed according to Bucić-Kojić et al. [12] with modifications. The mobile phases used consisted of water/formic acid/acetonitrile (87:10:3, *v/v*/*v*; mobile phase A) and water/formic acid/acetonitrile (40:10:50, *v/v*/*v*; mobile phase B). The gradient program was as follows: 10 min from 10 to 25% mobile phase B, 5 min from 25 to 31% mobile phase B, 5 min from 31 to 40% mobile phase B, 10 min from 40 to 50% mobile phase B, 10 min from 50 to 100% mobile phase B, 10 min from 100 to 10% mobile phase B. The sample injection volume was 20 μL and a flow rate of 0.8 mL/min.

#### 2.3.6. Determination of Individual Sugars

Extracts for the measurement of individual sugar concentrations were prepared by extracting 1 g of GP with 25 mL of distilled water in sealed bottles. Extraction was performed in a water bath with shaking at 170 rpm for 30 min at 30 °C. All extractions were performed in three replicates.

The individual sugar contents were analyzed by UHPLC using a refractive index detector (RID), according to the method attached to the column. Briefly, sucrose content was determined using an Aminex^®^ HPX column (HPX-87H, 300 × 7.8 mm, Bio-Rad Laboratories, Hercules, CA, USA) with a mobile phase of 5 mM of sulfuric acid and a flow rate of 0.6 mL/min at 40 °C for 60 min. The content of glucose, fructose, arabinose, and sucrose was determined using a Nucleogel^®^ Sugar Pb column (VA, 300 × 7.8 mm, Macherey-Nagel GmbH & Co. KG, Dueren, Germany), with HPLC grade water as the mobile phase and a flow rate of 0.4 mL/min at 80 °C for 20 min. Data were recorded and analyzed using the LabSolutions program (version 5.87). Sugars in the extracts were identified by comparing the retention time and spectral data with an authentic standard.

#### 2.3.7. Determination of Antioxidant Activity (AA)

The *AA* of the GPE was determined by DPPH, FRAP, and ABTS assays using a UV-VIS spectrophotometer (UV/VIS Spectrophotometer UV-1800, Shimadzu, Japan). Trolox (an analogue of vitamin E), which is a strong antioxidant, was used as positive control. All tests were performed in triplicate, and the final results were expressed in Trolox equivalents per dry basis of GP (g_TROLOX_/g_db_) (Equation (1)) as the mean of the replicates ± standard deviation (SD).

(1) $$AA({mg}_{TROLOX}/g_{db})=\frac{AA^{\prime }\cdot V}{m\cdot \left(\frac{w}{100}\right)}$$

where *AA′* is the antioxidant activity expressed as mg_TROLOX_/mL for each method, *V* is the volume of the extraction solvent (mL), *m* is the GP mass used for the extraction of the phenolic compounds (mg), and *w* is the dry matter content of GP (%).

The DPPH assay was performed according to the method of Bucić-Kojić et al. [37]. Briefly, 3.9 mL of DPPH-ethanol solution (0.026 mg_DPPH•_/mL) was added to 0.1 mL of sample, and the reaction mixture was incubated in the dark for 30 min. The absorbance at 515 nm was then measured relative to the blank sample (96% ethanol), and the inhibition of DPPH^•^ was calculated according to Equation (2):

(2) $${DPPH}_{inhibition}^{•}(\%)=\frac{A_{DPPH}-A_{s}}{A_{DPPH}}\cdot 100$$

where *A*_DPPH_ is the absorbance of the prepared DPPH-ethanol solution and *A*_S_ is the absorbance of the sample. The *AA′*_DPPH_ of samples was expressed as mg_TROLOX_/mL from the calibration curve obtained with Trolox, and the final results were expressed according to Equation (1).

The FRAP assay was performed according to the method of Benzie and Strain [38], with some modifications. Before analysis, the reagent FRAP was prepared consisting of 25 mL of 300 mM acetate buffer (pH 3.6) heated to 37 °C, 2.5 mL of 10 mM TPTZ solution (dissolved in 40 mM of HCl), and 2.5 mL of 20 mM FeCl_3_(H_2_O)_6_ solution. The samples for analysis were prepared by mixing 2.7 mL of FRAP reagent, 270 μL of distilled water and 150 μL of GPE, and the absorbance was read at 592 nm after 40 min of incubation in the dark at 37 °C. The blank sample was prepared in the same way, but distilled water was used instead of extract. The *AA*’_FRAP_ of the samples was expressed as mg_TROLOX_/mL from the calibration curve obtained with Trolox, and the final results were expressed according to Equation (1).

The ABTS assay was performed according to the method of Re et al. [39], but with modifications. Briefly, 950 µL of a diluted ABTS^•+^ radical solution was added to 50 µL of the extracts. The absorbance was measured after 10 min of incubation in the dark at 734 nm. The control sample was prepared in the same way, but ethanol was used instead of the sample. Absolute ethanol was used as a blank. After the measurement, the inhibition of ABTS^•+^ was calculated according to the following Equation (3):

(3) $${{ABTS}^{•+}}_{inhibition}\left(\%\right)=\frac{A_{0}-A_{s}}{A_{0}}\cdot 100$$

where *A*_0_ is the absorbance of the control sample and *A*_S_ is the absorbance of the sample. The *AA′*_ABTS_ of the samples was expressed as mg_TROLOX_/mL from the calibration curve obtained with Trolox, and the final results were expressed according to Equation (1).

### 2.4. Preparation of Phenol-Enriched Hydrogels

For the preparation of phenol-enriched hydrogels in the encapsulation process, a concentrated dry extract was used, which was prepared by drying the liquid GPE in a rotary evaporator at 48 mbar and 50 °C (Büchi, R-210, Flawil, Germany). The prepared phenol-rich dry extract was dissolved in a mixture of 30% aqueous ethanol solution and distilled water to a final concentration of 10 mg/mL. Specifically, 1.00 g of dry extract was dissolved in 20.8 mL of 30% aqueous ethanol solution, and 79.2 mL of distilled water was added after stirring on a magnetic stirrer for 60 min. This mixture was further stirred for about 120 min and then centrifuged at 15,000× *g* for 5 min to separate the undissolved extract particles. After centrifugation, 90 mL of the supernatant used for encapsulation was separated, and the remaining supernatant was used to determine the TPC (according to the method in Section 2.3.2.)

Encapsulation was achieved by SA, a combination of SA with GEL, and SA with GA as well as SA and CHIT. For the preparation of SA hydrogels, SA was added to the supernatant at a concentration of 3% (*w/v*). The mixture was stirred for 24 h to allow a so-called swelling, which allows homogeneity and better bonding of the coating with the desired active ingredient, but also to eliminate air bubbles that may form during the preparation of the alginate extract solution. After 24 h, encapsulation by ionic gelation was performed using an encapsulation device (Büchi B-390, Switzerland). In all experiments, a nozzle with a diameter of 450 µm, a frequency of 140 Hz, and an electrode voltage of 750 V was used. The operating pressure was adjusted during the encapsulations, as it depended on the viscosity and density of the coating and active ingredient mixture. Encapsulation was performed dropwise in 300 mL of 0.25 M CaCl_2_ to allow hydrogel formation. The hydrogels were stirred in CaCl_2_ for 10 min to allow complete solidification and then filtered onto filter paper. To remove calcium ions that were not crosslinked and remained on the surface of the hydrogels, the hydrogels were washed twice with 200 mL of distilled water. When GA and GEL were used as additional coatings, the same procedure was followed in their preparation, except that either GA or GEL was added to the mixture at a concentration of 1.6% (*w/v*) and 5% (*w/v*), respectively, along with sodium alginate (3%, *w/v*).

For the preparation of SA/CHIT hydrogels, SA hydrogels were first prepared according to the procedure described above, but after crosslinking in CaCl_2_, they were filtered and immersed in 1.5% (*w/v*) CHIT for 10 min with stirring. Then, the hydrogels were filtered and washed twice with 200 mL of distilled water. CHIT was prepared by dissolving for 24 h in 1% (*v/v*) glacial acetic acid to allow complete dissolution and homogeneous mixing.

Since the hydrogels were not stable enough, freeze-drying (Freeze-dryer Alpha 2-4 LSCplus, Christ, Germany) was performed to obtain microbeads with a longer shelf life. Before freeze-drying, the hydrogels were frozen at −80 °C (SWUF Ultra Low Temperature Smart Freezer, Witeg, Germany). The pressure during the freeze-drying process was 0.250 mbar and the drying time was 24–48 h, depending on the coating used.

### 2.5. Encapsulation Efficiency

After the hydrogels were prepared, the encapsulation efficiency (*EE*, %) was calculated from the TPC released into the crosslinking solution and washed off the surface of the hydrogels and the initial TPC in the extract involved in the encapsulation process using the following Equation (4):

(4) $$EE\left(\%\right)=\frac{C_{E}-C_{W}}{C_{E}}\cdot 100$$

where *C_E_* is the initial TPC measured in the supernatant to which the encapsulation coating(s) was further added, and *C_W_* is the TPC found in calcium chloride and wash water combined. The results are expressed as the mean value of the replicates ± standard deviation (SD).

### 2.6. Characterization of Dried Microbeads

#### 2.6.1. Size, Shape, and Texture of Microbeads

The geometric characteristics (size and shape) of hydrogels and dry microbeads were studied by computer image analysis. The EPSON V500 Photo Scanner (Epson America Inc., Long Beach, CA, USA) was used to capture and digitize the samples with a resolution of 800 dpi, color depth of 24 bits in sRGB model, and format TIFF. Exactly 10 hydrogels/dry microbeads were stacked on a glass Petri dish, taking care that they did not touch each other. Then, the Petri dish containing the samples was placed on the scanner, which was located in a dark chamber to eliminate the influence of external light and reduce the possibility of errors during scanning. After scanning, the images were processed using the ImageJ program (version 1.59g, Wayne Rasband, NIMH, Bethesda, MD, USA). Parameters divided into two groups were read-parameters of size (area, perimeter, max and min Feret diameter) and shape (circularity, roundness, solidity). The results of the measurement of the size of the samples were converted from pixels to millimeters, taking into account the known values of the scanner resolution in dpi units. The shape parameters provide information on how much the shape of the analyzed sample deviates from a regular circle. The values range from 0 to 1, where 1 indicates the value of a perfect circle (Table 1).

The shape parameters were calculated using software ImageJ User Guide-IJ 1.46r [40]. All measurements were performed in triplicate, taken from different batches, and expressed as the mean of the measurements ± SD.

The texture profile analysis of the hydrogel beads was performed using the TA.XTplus Texture Analyzer (Stable Microsystems Ltd., Surrey, UK). The single hy-drogel/freeze-dried microbead was subjected to a double compression of 50% with a delay of 2 s between compressions and a test speed of 0.5 mm/s. An aluminum cylinder probe with a diameter of 10 mm was used. The hardness value, determined from the texture profile analysis curves, was read after 2 s of compression at the point on the curve where the bead was compressed to 1 mm. Individual dried microbeads were compressed with the same probe to a compression load of 20% at a test speed of 0.1 mm, and the hardness (N) was determined as the maximum peak height. The texture of ten hydrogel/freeze-dried microbeads from each sample was evaluated and expressed as the mean of the measurements ± SD.

#### 2.6.2. Scanning Electron Microscopy (SEM)

The morphology of the prepared freeze-dried microbeads was examined by scanning electron microscopy (Hitachi S4700, Hitachi Scientific Ltd., Tokyo, Japan) at 10 kV. The samples were previously coated with a thin gold–palladium film using a sputter coater (Bio-Rad SC 502, VG Microtech, Uckfield, UK).

#### 2.6.3. X-ray Powder Diffraction (XRPD)

The crystalline structure of the extract, coatings and freeze-dried microparticles, was characterized using an X-ray powder diffraction system (BRUKER D8 Advance diffractometer, Karlsruhe, Germany). The samples were measured with Cu Kα radiation (λ = 1.5406 Å) and scanned at 40 kV and 40 mA in the interval of 3–40 2θ with a VÅNTEC-1 detector. The further evaluation of results, smoothing, Kα2-stripping, and background removal were performed using DIFFRAC plus EVA software (Karlsruhe, Germany). Prior to the measurement, freeze-dried microbeads were pulverized using a mortar and pestle.

#### 2.6.4. Differential Scanning Calorimetry (DSC)

To evaluate the thermal behavior of produced microbeads, extracts, and coatings, differential scanning calorimetry (Mettler Toledo 821e DSC; Mettler Inc., Schwerzenbach, Switzerland) was applied. Approximately 3–5 mg of the samples was measured in the temperature interval of 25–300 °C at a heating rate of 10 °C/min, under a constant argon flow of 150 mL/min.

### 2.7. In Vitro Release of Phenolic Compounds

The in vitro release of phenolic compounds from the prepared dry microbeads was performed according to the protocol described by Minekus et al. [41] with modifications.

Briefly, the release consisted of three phases: oral, gastric, and intestinal. Electrolyte solutions mimicking those of the human gastrointestinal tract were used for each of these phases, with release occurring without the use of enzymes. A temperature of 37 °C was maintained throughout the process (243 min in total) and stirring was performed with a magnetic stirrer. To the dry microbeads (100 mg), 4 mL of simulated salivary fluid (SSF) and 25 μL of CaCl_2_ (H_2_O)_2_ were added. The pH was then adjusted to 7, and redistilled water was added to a total volume of 10 mL. After 3 min, 2 mL of the sample was removed for further analysis of the phenolic compound content, and 2 mL of the SSF solution was returned to the system. After a three-minute oral phase, the gastric phase was initiated by adding 8 mL of simulated gastric fluid (SGF) and 5 μL of CaCl_2_ (H_2_O)_2_. The pH was then adjusted to 3 with 1 M of HCl to fully simulate the acidic conditions prevailing in the stomach. After reaching the desired pH, water was added to a total volume of 20 mL. This phase lasted for 120 min, during which aliquots of the sample were taken at specific time intervals and the same volume of simulated intestinal fluid (SGF) was returned to the system, as described in the oral phase. At the end, 16 mL of the SIF solution and 40 μL CaCl_2_ (H_2_O)_2_ were added. The pH was then adjusted to 7 and redistilled water added to a final volume of 40 mL. The sampling procedure was the same as for the previous two phases, except that the SIF solution was returned to the system. This phase also lasted 120 min.

### 2.8. Release Kinetics of Phenolic Compounds from Microbeads

Method-dependent and mathematical function-based methods are extremely useful to describe the release of bioactive components from an encapsulated system. Considering this fact, four mathematical models: the first order model, the Higuchi model, the Hixson–Crowell model, and the Korsmeyer–Peppas model, were used to describe how the release of phenolic components from phenol-enriched dry microbeads occurs in the gastrointestinal tract during simulated digestion in vitro. Data were obtained using DDSolver [42], and the characteristics of the model are summarized in Table 2. Three criteria were used to determine which model best described the release of phenolic compounds, namely the adjusted coefficient of determination (R^2^adj), the Akaike information criterion (AIC), and the model selection criterion (MSC).

## 3. Results and Discussion

### 3.1. Chemical Composition of the Grape Pomace

GP includes the seeds, pulp, skins of the grapes and sometimes the stems, as well as some other solid components that remain after extraction by pressing the grapes. Each of these components of GP is rich in certain compounds. Thus, the skin of the grapes contains many tannins and anthocyanins; the seeds are rich in flavan-3-ols, fibers, lipids and proteins; while the stems contain cellulose and hemicellulose. The chemical composition of GP depends on several factors, such as geographic origin, climate, harvest time and grape varieties, but also on the degree of ripeness at the time of harvest. As shown in Table 3, the studied GP is rich in numerous components, which is why it can be used in biorefineries to produce high-value products (biofuels, enzymes, compost, functional foods, etc.) while reducing the negative impact on the environment, which is the core of the circular bioeconomy.

The content of ash (4.89%_db_), free fats (8.18%_db_), and crude protein (7.38%_db_) is an indicator of the content of minerals and amino acids in the samples used. Deng et al. [52] studied the GP composition of the Cabernet Sauvignon variety and found that the percentage of crude proteins (12.34%_db_) and ash (7.59%_db_) was higher than in this study. Nevertheless, the content of free fats is 29.2% higher than the value published by Deng et al. [52]. Dietary fats are considered a source of energy and contribute to the functional and sensory properties of foods, such as smoothness and palatability. They are also carriers for many other compounds, such as flavors, vitamins, and colors, which contributes to the potential use of GP for animal feed production [53]. GP is a lignocellulosic material, indicated in this study by a certain amount of insoluble fibers such as lignin (25.80%_db_), cellulose (14.22%_db_), and hemicellulose (10.31%_db_), which, due to their low density and high porosity, can improve digestion by increasing stool volume and accelerating intestinal transit [54]. These fibers have a non-negligible number of phenolic compounds entrapped in their structure. The release of these compounds from the complex lignocellulosic structure can improve the biological activity of GPE, for which numerous pretreatment methods for lignocellulosic material and extraction methods have been developed [55]. From all this, it is evident that GP has a high nutritional value and can be used not only for the production of animal feed, but also for food fortification [56].

Of the eleven sugar standards tested, four were quantified in the GPE: glucose (4.53 mg/g_db_), arabinose (1.35 mg/g_db_), sucrose (3.23 mg/g_db_), and fructose (8.51 mg/g_db_). In addition, GP is often the subject of research for ethanol and biofuel production due to its high sugar content [57]. For biofuel production, the ratio of total carbon to total nitrogen is important. The results of the analysis of total carbon in solid GP (1.27%_db_), and liquid extract (35.63%_db_) and total nitrogen (1.27%_db_), shed light on the potential use of Cabernet Sauvignon GP for the production of biofuels as well as biofertilizers through the pyrolysis process [54].

Phenolic compounds are of great interest to many industries such as pharmaceuticals, food, cosmetics, and many more. As can be seen in the results presented in Table 3, the GP variety used in this study has the highest content of TPC (53.55 mg/g_db_) followed by TFC (22.85 mg/g_db_) and TPA (9.79 mg/g_db_). Deng et al. [52] studied the extraction of phenolic compounds from Cabernet Sauvignon GP using two extraction methods (ultrasonic extraction in bath and extraction in environmental shaker) and two solvent mixtures (0.1% HCl/70% acetone/29.9% H_2_O and 70% acetone/30% H_2_O) and obtained higher values for TFC and TPA than in this work, while lower values for TPC were obtained. On the other hand, in their study, Šelo et al. [6] used the same extraction method as in this study, also from the Cabernet Sauvignon variety (Erdut vineyards, Croatia, harvest 2016), and obtained lower values for TPC and TPA. These data show that not only the extraction conditions, but also the agronomic and geographical conditions, as well as the harvest time, have a great influence on the phenolic compound content in the GPE.

The antioxidant activity of natural extracts is often associated with the presence of phenolic compounds. In this study, the antioxidant activity was tested by the DPPH and ABTS methods, and the reducing power by the FRAP method (Table 4).

The ABTS method produced much higher values for the antioxidant activity of GPE (313.83 mg_TROLOX_/g_db_) than the DPPH method (70.46 mg_TROLOX_/g_db_). Rockenbach et al. [58] obtained exactly opposite results for the Cabernet Sauvignon variety, finding higher antioxidant activity with the DPPH method (126.83 mg_TROLOX_/g_db_) than with the ABTS method (121.79 mg_TROLOX_/g_db_). Moreover, in Rockenbach’s study, of the four GP varieties tested (Cabernet Sauvignon, Merlot, Bordeaux, and Isabel), the Cabernet Sauvignon variety had not only the highest antioxidant activity, but also the highest reducing power tested by the FRAP method, namely 62.34 mg_TROLOX_/g_db_, while in this study it was 78.34 mg_TROLOX_/g_db_.

The slightly higher values of TPC compared to TFC and TPA can be explained by the fact that the Folin–Ciocalteu method is not only specific for polyphenolic compounds, but also detects all phenolic groups present in the extracts, including extractable proteins. In addition, the method is sensitive to the presence of interfering substances (e.g., ascorbic acid, saccharides), which also reduce the Folin–Ciocalteu reagent as well as the polyphenolic compounds [59]. Therefore, a profile of the individual phenols was also established.

The UHPLC analysis showed that GP is rich in many individual phenolic compounds (Table 5). Ten phenolic acids were quantified, of which gallic acid stood out with the highest concentration (383.41 μg/g_db_), which is in agreement with the results of Iora et al. [60] and Rockenbach et al. [58].

Numerous in vitro and in vivo studies have shown that gallic acid and its derivatives have beneficial effects in the prevention of lung, colon, and breast cancer, among others [61]. After gallic acid, ellagic acid was found in high concentrations (127.34 μg/g_db_). Ellagic acid not only has antioxidant and anti-inflammatory effects [62], but also antihyperlipidemic and antihyperglycemic properties, which have been confirmed by in vitro and in vivo studies [60,61]. In addition, it has been shown to be effective in the treatment of bladder [63], colon [64], and breast [65] cancer. Other phenolic acids found in GP also have beneficial effects on human health. Thus, Altay et al. [66] found that extracts from *Gysophila* species rich in vanillic, 3,4-dihydroxybenzoic, *p*-hydroxybenzoic, *p*-coumaric, and syringic acids can inhibit the proliferation of liver, colon, and breast cancer cells.

In GPE, flavan-3-ols predominate, of which catechin (3088.84 μg/g_db_) and epicatechin (1279.11 μg/g_db_) have the highest content. The beneficial effects of epicatechin (anti-inflammatory, anticarcinogenic, antidiabetic, and antimicrobial) have been widely researched and described [67]. These compounds are present in the largest amounts in the skin and seeds of grapes [68]. In addition, grape seeds are a known source of proanthocyanidins. The obtained results are in agreement with those of de Sa et al. [69], i.e., after catechin and epicatechin, high levels of procyanidins B1 and B2 were found. Epigallocatechin gallate and gallocatechin gallate were also quantified in GPE. The results show that the concentration of gallocatechin gallate is almost 6.45 times higher than that of epigallocatechin gallate, which is due to the fact that it is chemically much more stable than epigallocatechin gallate. These properties allow the use of gallocatechin gallate in the preparation of creams to protect the skin from UVB radiation [70]. Among the studied flavonols, quercetin stands out (470.69 μg/g_db_), which has been attributed to its potential use in the treatment of eye diseases [71], as an antiviral agent [72], etc. In addition, all of the quantified flavanols, quercetin, kaempferol, and rutin, have been shown to be phenolic compounds that act as cholinesterase inhibitors; therefore, they may have potential use in the treatment of Alzheimer’s disease [73].

Two stilbenes were identified in GPE with *ε*-viniferin found at higher concentration (62.27 μg/g_db_) than resveratrol (29.82 μg/g_db_). Resveratrol has been intensively studied for its anticancer effects in lung [74], skin [75], colon [76], ovary [77], prostate [78], and many more. It has also been found that its dimer, *ε*-viniferin, plays an important role in the inhibition and also progression of cancer [79].

Anthocyanins are a subgroup of flavonoids mainly found in the skin of grapes and are responsible for the color of red grapes. They are very sensitive to changes under the influence of light, oxygen, temperature fluctuations, and pH. Oenin chloride (509.16 μg/g_db_), peonidin-3-*O*-glucoside chloride (63.54 μg/g_db_), and myrtillin chloride (42.05 μg/g_db_) were detected in the largest amounts. Oenin plays a role in controlling long- and short-term cellular activities, and studies have also shown that it can inhibit tumor cells such as gastric adenocarcinoma, colon cancer, and promyelocytic/monocytic leukemia [80,81,82]. Sari et al. [83] studied the anthocyanins of black rice and found that myritillin and peonidin-3-*O*-glucoside may have a biological function as inhibitors of an inflammatory cytokine important in the treatment of metabolic diseases associated with obesity.

Overall, it appears from this paper, as well as other available literature, that GP is rich in phenolic compounds that may contribute to health benefits through various mechanisms. The beneficial effects of phenolic compounds on human health are highly dependent on them reaching a target site in the body where they can be absorbed, i.e., the intestine. Phenolic compounds are extensively metabolized during food intake, resulting in the formation of various metabolites, and the variability of the beneficial response depends on the form of phenolic compounds consumed [84]. In the following, the focus of this work is on encapsulation and its influence on the controlled release of phenolic compounds during simulated digestion in vitro.

### 3.2. Encapsulation Efficiency of Total Phenolic Compounds from Grape Pomace Extract

The *EE* of TPC from GPE in different alginate-based microbeads was determined, which showed that it is possible to influence the efficiency by adding different coatings of natural origin to SA.

**Figure 1:**
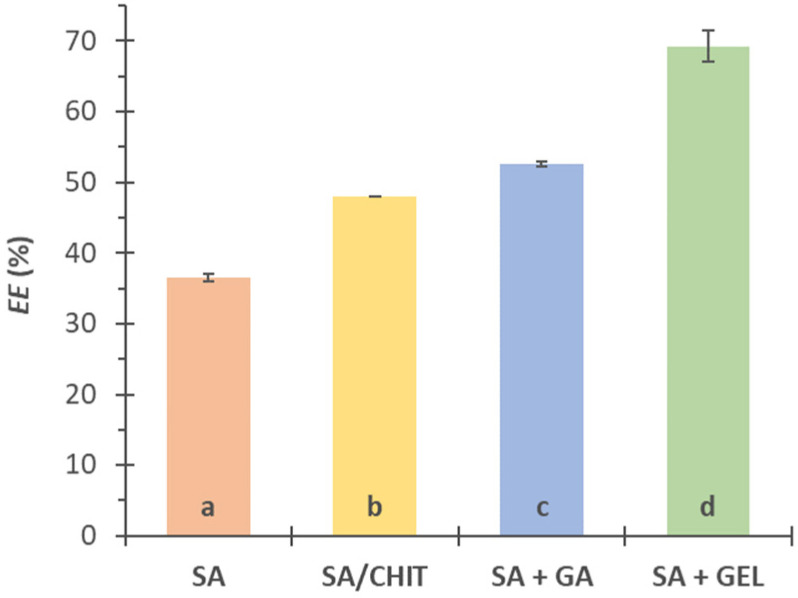
Encapsulation efficiency (*EE*, %) of phenol-rich grape pomace extracts using various coatings (3% *w/v* sodium alginate, SA; combination SA with 1.5% chitosan, SA/CHIT; SA with 1.6% *w/v* gum arabic, SA + GA; and SA with 5% *w/v* gelatin, SA + GEL). Different letters represent significant differences (ANOVA, Duncan’s test at *p* < 0.05).

The *EE* using sodium alginate was 36.54%, while the addition of chitosan, gum arabic, and gelatin increased *EE* to 48.04%, 52.62%, and 69.27%, respectively (Figure 1). The ANOVA test showed that there is a statistically significant difference (Duncan’s test, *p* < 0.05) between *EE*s reached by different applied coatings. Due to its cationic nature, CHIT forms a membrane around the hydrogel of polyanionic alginate by forming bonds between the carboxylate groups of the alginate and the protonated amino groups of the chitosan. Due to these bonds, fewer phenolic compounds are lost during encapsulation and the produced beads are firmer. The addition of coatings such as GA reduces the porosity of the SA gel, preventing the leakage of phenolic compounds from the microbeads during ionic gelation. Moreover, the proteins contained in the structure of GA can interact with the phenolic compounds from the GPE, which also provides better binding. Proteins are known to interact with polyphenolic compounds to form hydrophobic and hydrogen bonds, but they can also bind with polymers with free carboxyl groups. This explains the great increase in *EE* by combining gelatin with sodium alginate [85,86].

### 3.3. Physicochemical Characterization of Microbeads

#### 3.3.1. Size, Shape, and Texture

The effect of applied coatings and freeze-drying on the size parameters (area, perimeter, maximum and minimum Feret values), shape parameters (roundness, circularity, and solidity), and texture (hardness) of the microbeads was studied (Table 6). Microbeads based on pure SA had the lowest values for all tested size parameters compared to other microbeads. After drying, the largest change was observed in SA hydrogels, where area, perimeter, Feret_MAX_, and Feret_MIN_ decreased by 61.99%, 35.12%, 33.02%, and 40.55%, respectively. Hydrogels prepared with the addition of GEL, GA, and CHIT had approximately similar values for min and max Feret, while according to the data for area (16.56 mm) and perimeter (16.09 mm), hydrogels with GEL were the largest. After drying, the area of microbeads with CHIT decreased significantly (44.34%), while the values for microbeads with GEL and GA were almost the same for all size parameters, although a larger decrease was observed for SA + GEL microbeads. Compared to hydrogels, a 38.04% reduction in area was observed for SA + GEL microbeads, while this was 30.73% for SA + GA microbeads. Moreover, the perimeter of SA + GA microbeads decreased the least during drying (7.17%).

**Figure 2:**
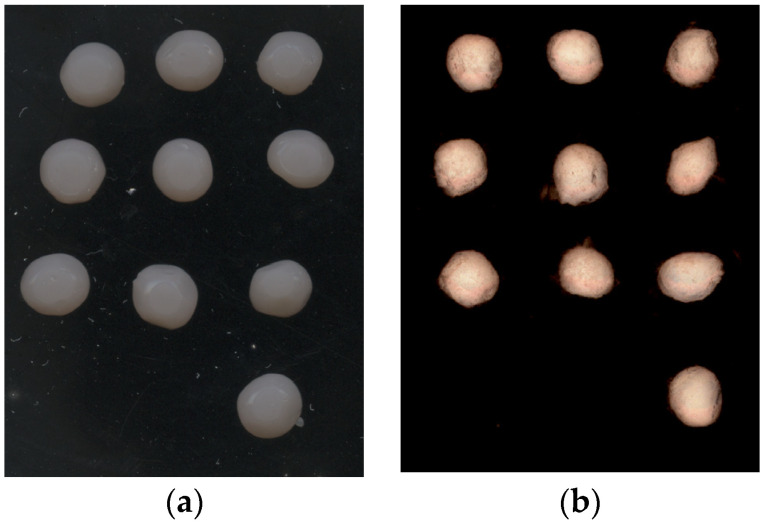
(**a**) Hydrogels and (**b**) freeze-dried microbeads prepared with sodium alginate and gelatin.

The results for the shape of the hydrogel show that SA microbeads lost their spherical shape, as evidenced by an 11.30% decrease in roundness and a 10.92% decrease in circularity after freeze-drying. The greatest changes due to drying were observed in circularity, especially for SA + GA microbeads, whose reduction was 18.69% compared to hydrogels. The solidity, which can also be described as surface roughness, ranged from 0.94 to 0.96 for dried microbeads, and the closer the value was to 1, the smoother the surface. As expected, hydrogels had higher values for this parameter than freeze-dried microbeads because, after their production, it can be seen (an example for SA + GEL microbeads is shown) that they had a taut surface (Figure 2).

It can be seen from Table 6 that the hardness of the hydrogels changed significantly after freeze-drying. The largest change is seen in SA microbeads, where the hardness increased by 88.31%, and the smallest change is seen in SA/CHIT microbeads compared to hydrogels (24.07%). It is also evident that SA hydrogels had the highest hardness (0.398 N) and that the addition of other coatings decreased the hardness, which is consistent with the results of Zamora-Vega et al. [87] and Sandoval Mosqueda et al. [88]. The addition of other coatings led to softer hydrogels, and the strength was reduced because there was no strong molecular interaction between the constituents in the hydrogels.

#### 3.3.2. Scanning Electron Microscopy Analysis

**Figure 3:**
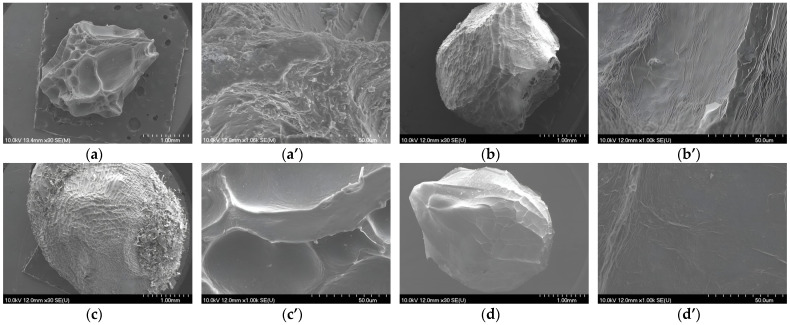
SEM images of freeze-dried microbeads of sodium alginate (**a**,**a′**); sodium alginate with gum arabic (**b**,**b′**), with gelatin (**c**,**c′**) and with chitosan (**d**,**d′**); and their outer layer with the scale bar of 1 mm and 50 µm for each sample.

The effect of the coatings used to encapsulate the phenol-rich extract of GP on the morphology of the microbeads was studied using SEM (Figure 3). SEM reveals wrinkles, cracks, pores, and inclusions on the surface of the obtained microbeads. Microbeads prepared with SA as a coating are visibly smaller than those with additional coating (Figure 3a,a’). It can also be seen that the surface structure of the SA microbeads was wrinkled and rough with many protrusions, such as was described by Li et al. [89]. When calcium ions encounter the surface of a droplet containing SA, crosslinking occurs exclusively at the surface of the forming hydrogel and the polymer chains are closer together. This results in a less permeable surface that prevents calcium ions from reaching the interior of the hydrogels. Such a surface full of cross-linked calcium ions explains the rough structure of the dried microbeads [90]. In addition, the microbeads with SA lost the spherical shape they had before by drying, but it is obvious that the alginate microbeads to which an additional coating was added kept this shape. During freezing, ice crystals are formed, which causes local expansion in the microbeads, revealing pores and cavities on their surface [91]. Such voids were visible in the microbeads to which GEL was added (Figure 3c,c’).

Ferreira Almeida and Almeida [92] also observed this when pindolol was encapsulated in alginate–gelatin microbeads and found that the addition of formaldehyde as a crosslinking agent made the surface of these microbeads smoother. Microbeads with SA/CHIT appeared to have the smoothest structure without many pores (Figure 3d,d’), as reported by Xing et al. [93], while Li et al. [89] reported that the alginate microbeads with chitosan had a rough and coarse surface. The combination of SA and GA as coating materials resulted in microbeads that were not completely spherical and had a rough texture, as shown in Figure 3b,b’. The uneven surface of the microbeads was also visible, as observed by Chun et al. [94] and Sandoval Mosqueda et al. [88], when encapsulating probiotic cells with a combination of these two coatings. Cracks on the surface are caused by the partial breakdown of the polymeric gel network structure during freeze-drying, but some research has shown that the use of natural polysaccharides or proteins can partially overcome this problem [91,95]. Thus, by adding a sufficient concentration of the additional coating, it is possible to preserve the structure of the microbead and prevent excessive destruction of the polymer structure of the coating. This is evident in this work compared to that of Li et al. [89]. In particular, the structure of the microbeads in the SA + GA and SA/CHIT combinations is visibly different in terms of surface structure. Li et al. [96] found that the microbeads with this combination of coatings were irregularly shaped and had a distinctly wrinkled surface, whereas the microbeads in this work were almost spherical and had a smoother surface. A morphological analysis of the surface of microbeads is very important because knowledge of the surface properties can help clarify the release mechanism of the encapsulated active ingredients. That is, the greater the number of pores, the greater the likelihood that the solvent will encounter the interior of the particle and assist in the release of the active ingredient.

#### 3.3.3. Differential Scanning Calorimetry and X-ray Powder Diffraction Analyses

Data on the structural organization of the extract and polymer coatings used to prepare the microbeads were obtained by DSC and XRPD.

**Figure 4:**
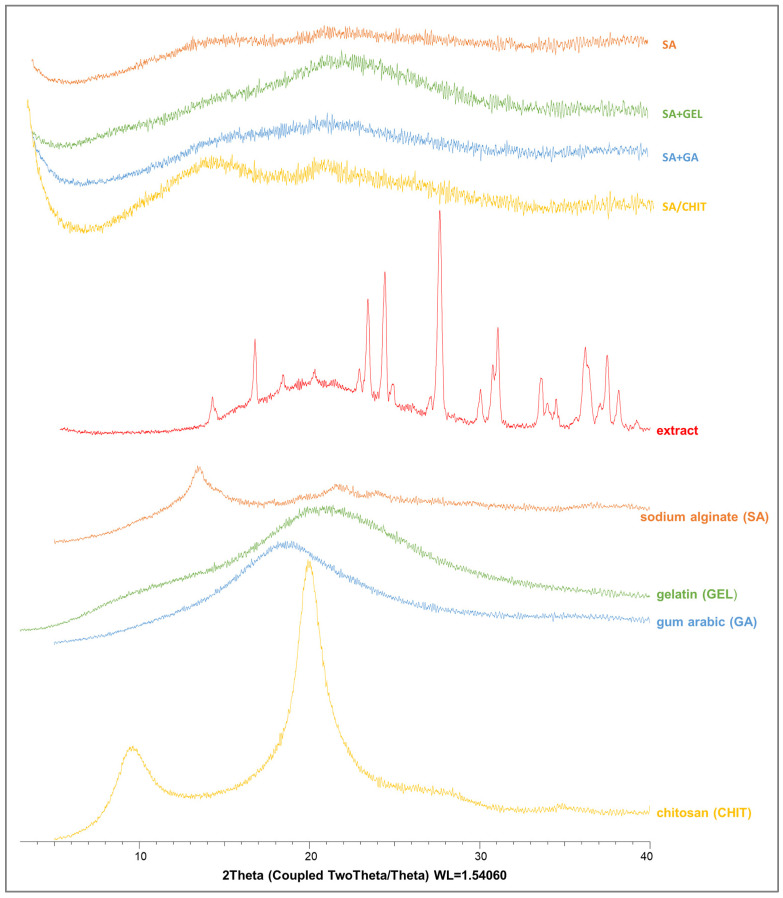
X-ray powder diffractograms of grape pomace extracts, pure coatings (sodium alginate, SA; chitosan, CHIT; gum arabic, GA; and gelatin, GEL), and freeze-dried microbeads containing phenol-rich grape pomace extract prepared using various coatings (SA; combination SA/CHIT; SA + GA; and SA + GEL).

During encapsulation, a change in the crystalline structure of the active compound is possible; in most cases it is an amorphization, which has been confirmed in many articles [97,98,99,100]. In the XRPD analysis, the GPE showed peaks at 2θ from 14° to 39°, indicating a crystalline structure (Figure 4). However, the coatings used for encapsulation did not show crystalline peaks, implying the amorphous nature. The results show that there were no peaks of the extract in the freeze-dried microbeads, indicating that the GPE was dispersed at the molecular level in the microbeads during the encapsulation process.

**Figure 5:**
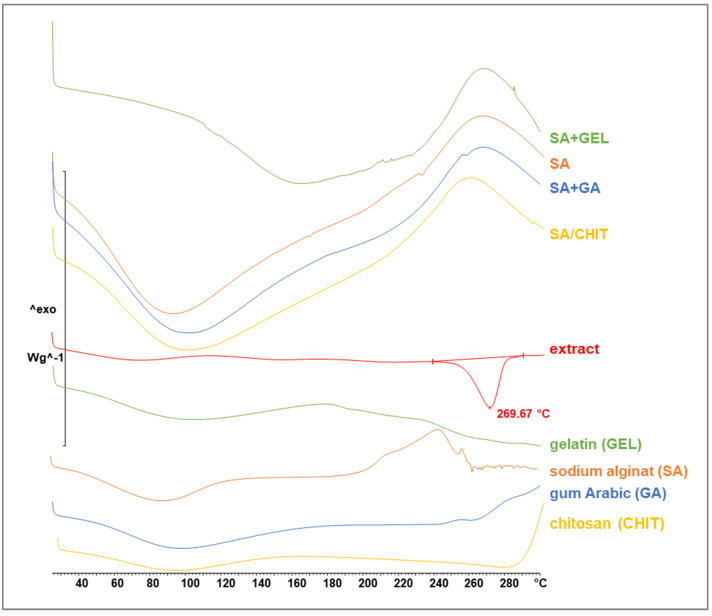
Differential scanning calorimetry thermograms of grape pomace extracts, pure coatings (sodium alginate, SA; chitosan, CHIT; gum arabic, GA; and gelatin, GEL), and freeze-dried microbeads containing phenol-rich grape pomace extract prepared using various coatings (SA; combination SA/CHIT; SA + GA; and SA + GEL).

Figure 5 shows the DSC curves, indicating that the GPE exhibited an endothermic transition at 269.67 °C, which corresponds to melting, but also confirms the XRPD patterns regarding the crystalline structure of the GPE. Precisely because of its crystallinity, GPE did not completely dissolve during preparation for encapsulation, and its crystallinity makes GPE insufficiently soluble and less accessible for passage across the intestinal membrane and subsequent absorption [101,102]. The DSC curves show that the crystalline structure of the extract turned into an amorphous structure when SA, GA, GEL, and CHIT were used as the coating for encapsulation. The broad peaks in the DSC curves of the encapsulated extract are due to the water loss caused by the high temperatures.

### 3.4. In Vitro Release of Phenolic Compounds from Encapsulated Grape Pomace Extracts

The release of phenolic compounds from the freeze-dried microbeads occurred in three phases: oral, gastric, and intestinal, using enzyme-free electrolyte solutions that simulate conditions in the human gastrointestinal tract. The formation of pores in the alginate network has a major impact on the release of bioactive components from the microbeads, and the addition of other coatings can both influence the filling of these pores and allow a delayed release [103]. GA, CHIT, and GEL were added to the SA to create a system that allows the phenolic compounds to avoid destruction in the oral and gastric phases, while allowing them to be safely transported and slowly released in the intestinal phase. The intestine is the preferred site for the release of phenolic compounds, as it provides a greater absorption opportunity and allows the execution of desirable properties such as anti-inflammatory, anti-carcinogenic, antimicrobial, and many others [3].

**Figure 6:**
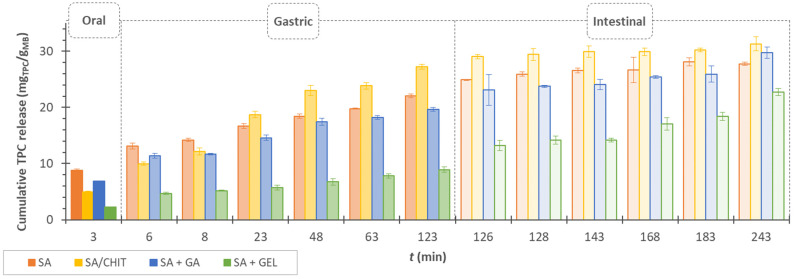
Cumulative release of total phenolic compounds (TPC) from freeze-dried microbeads (MB) containing grape pomace extracts prepared with sodium alginate (SA), sodium alginate with chitosan (SA/CHIT), sodium alginate with gum arabic (SA + GA), and sodium alginate with gelatin (SA + GEL).

Performing the release in solutions, the cumulative release rate in the oral phase ranged from 17.49% (SA + GEL) to 31.79% (SA) (Figure 6).

In this short phase, rapid diffusion of the phenolic compounds into the surrounding solution occurs, as well as the immediate release of the phenolic compounds on the surface of the microbeads. The diffusion then continues during the gastric phase. During the release in the gastric phase and the change of pH from neutral to acidic, the color of the electrolyte solution containing the microbeads changes to an intense red, which is due to the anthocyanins in the grape pomace extract. During the 120 min gastric phase, the microbeads retain a spherical shape with all coatings used. After the beads have passed to the intestinal phase, the color of the solution turns brown, indicating the decomposition of the pigments present.

The beads also gradually decompose because the sodium ions present in the SIF solution exchange with divalent calcium ions and electrostatic repulsion occurs between the negative charges of the COO- groups in the alginate structure. This causes the chains to relax; the “egg box” structure of the microbeads is destroyed and they disintegrate [104].

During the release of phenolic compounds in the gastric phase, 47.54% of phenolic compounds were released from the SA microbeads, and another 20.67% were released in the intestinal phase (Figure 6). According to Stoica et al. [105], of encapsulated phenolic compounds extracted from rose hips with SA and a combination of SA and CHIT, 40.7% of phenolic compounds were released in SGF and another 3.7% in SIF from SA beads. Meanwhile, using 1% CHIT mixed with CaCl_2_ with SA resulted in a release of slightly less than 80% in SGF and 15.4% in SIF. Otherwise, in this work, after the preparation of SA microbeads, 1.5% (*w/v*) CHIT was used as the medium in which the obtained alginate microbeads were immersed for additional coating. During the release, 59.29% of phenolic compounds were released in SGF and another 15.25% in SIF (Figure 6). On the other hand, Yousefi et al. [106] encapsulated the extract of *Viola odorata* L. extract with SA and CHIT (1% *w/v*) using the emulsification method followed by external gelation, concluding through in vitro digestion simulations that fewer compounds were released in SGF and more in the intestinal phase. These results suggest that the method of addition of CHIT, as well as its concentration, affects the release of bioactive compounds from the prepared beads. Overall, the microbeads prepared with SA/CHIT as an additional coating had the highest release rate of phenolic compounds. The most likely reason for this is that in an acidic medium, the amino groups of the chitosan are ionized and the hydroxyl groups of the phenolic compounds are deprotonated, leading to the swelling of the beads and allowing the release of the compounds [107,108].

GA is an ampholytic polymer that attracts negatively charged alginate molecules by electrostatic forces. Calcium ions also react with the carboxylate groups of GA. Tsai et al. [109] encapsulated phenolic compounds from radish juice byproducts using SA and GA as a coating by the reverse spherification method. By performing in vitro digestion simulation in SGF and SIF, the results showed that the phenolic compounds were released faster in SIF than in SGF. By studying the addition of different concentrations of GA in combination with SA, they concluded that the concentration strongly affected the preservation of phenolic compounds in SGF. According to the results of this work, 66.05% of phenolic compounds were released from the microbeads prepared by SA + GA at the end of the gastric phase, of which 22.99% had already been released in SSF (Figure 6). Therefore, in further studies, it might be necessary to adjust the concentration of GA to achieve a higher retention of phenolic compounds during SGF.

A large difference was observed in the release tendency of phenolic compounds from the SA + GEL microbeads. All samples followed the same release trend, an increased release in SGF and a decrease in SIF, except SA + GEL (Figure 6). The addition of gelatin allowed the release of 41.55% in SSF and SGF together, while the remaining 58.45% of phenolic compounds were released in the intestinal phase, i.e., at the most desirable site. Da Silva Carvalho et al. [110] encapsulated an anthocyanin-rich extract in alginate beads and immersed them in GEL. Upon release in the stomach, up to 70% of anthocyanins were released from these beads.

Assuming that the release of phenolic compounds from the microbeads is related to their morphological characteristics, the higher release from SA and SA + GA microbeads suggests the presence of very small pores seen in the SEM images that allow the penetration of digestive solutions into the microbeads. These pores are not seen in SA/CHIT microbeads and yet high amounts of phenolic compounds are released from these microbeads. There is a possibility that they would be noticeable at a different magnification during SEM analysis, but as mentioned earlier, the high release from SA/CHIT microbeads could be due to the swelling of the microbeads in acidic medium after ionization of the hydroxyl groups of chitosan. The microbeads SA + GEL have a textured surface and many depressions, but no pores are visible at a higher magnification when analyzed by SEM. This type of release can also be related to *EE*, i.e., a higher *EE* is caused by the fact that phenolic compounds are well bound to proteins, so bound phenolic compounds need more time to be released from the network of gelatin and sodium alginate.

### 3.5. Release Kinetics of Phenolic Compounds from Microbeads

Modeling the release kinetics of active compounds is of great importance because mathematical models facilitate the determination of important physical parameters (e.g., the diffusion coefficient of the active compound), and it is also possible to predict the behavior of the encapsulated agent during release before release occurs. In this way, the coating(s) used, the active ingredient, and the size and shape of the encapsulated particles can be taken into account and the release profile theoretically predicted [111]. In this study, the models listed in Table 2 (the first-order model, Hixson–Crowell model, Higuchi model, and Korsmeyer–Peppas model) were tested to describe the mechanism and kinetics of the release of phenolic compounds from microbeads. The parameters of the kinetic models describing the release of phenolic compounds from the microparticles and the statistical criteria for the success of the model approximation are listed in Table 7.

When comparing a model with a different number of parameters, it is necessary to use an adjusted coefficient of determination (R^2^adj), because the coefficient of determination (R^2^) increases with the number of parameters and R^2^adj may decrease if overfitted. For this reason, the model with the largest R^2^adj is considered the best model for fitting the experimental data [43]. On the other hand, if two or more models are compared, the model with the lower AIC value is considered the appropriate model. This criterion is commonly used and is an excellent method to determine which model describes the release of any agent. MSC is another popular statistical criterion that attracts attention when selecting a model for in vitro release. It is a modified reciprocal form of AIC, and the most appropriate model for comparison is the one with the highest MSC value.

According to the statistical criteria for the success of the approximation of the experimental data (Table 7), it can be seen that the release of phenolic compounds from the microbeads coated with SA, SA/CHIT, and SA + GA follows the Korsmeyer–Peppas model (R^2^adj = 0.966 − 0.973; MSC = 2.751 − 2.939; AIC = 84.141 − 95.693).

**Figure 7:**
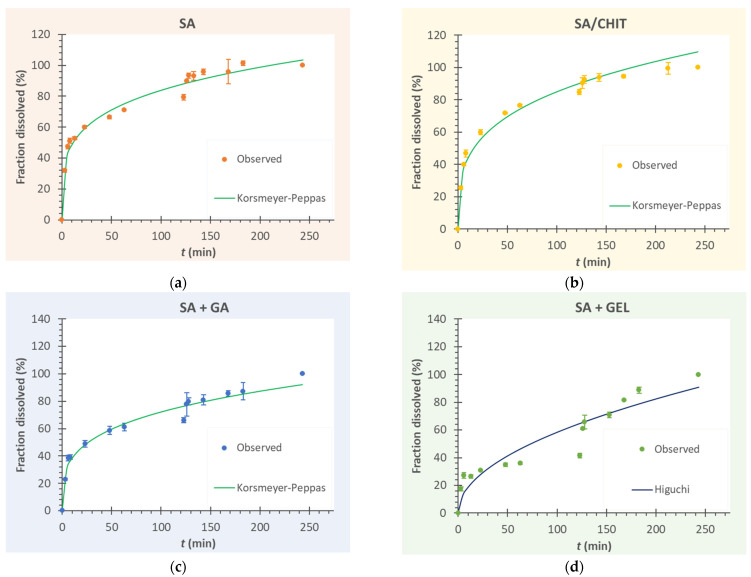
Kinetics of phenolic compound release from freeze-dried microbeads containing grape pomace extracts prepared with (**a**) sodium alginate (SA); (**b**) sodium alginate with chitosan (SA/CHIT); (**c**) sodium alginate with gum arabic (SA + GA); and (**d**) sodium alginate with gelatin (SA + GEL) (symbols—experimental data, lines—approximate curves, according to the most suitable model).

The best model for the individual freeze-dried microbeads is shown in Figure 7a–c (all results are included in the Supplementary Material). The *k*_KP_ parameter of the Korsmeyer–Peppas model provides information about the ionic strength of the surrounding solutions, i.e., the environment in which the microbeads are located. The smaller the value of *k*_KP_, the higher the ionic strength in the external environment, which can affect the release of the active compounds by affecting the microbeads structure and bonding of the coating(s) with the phenolic compounds. In short, the higher the ionic strength, i.e., the lower the *k*_KP_ value, the greater the shrinkage of the microbeads may occur [112]. In addition, a higher *k*_KP_ value indicates a faster release of active ingredients, which was observed in SA microbeads. In other words, the highest cumulative release was recorded when 31.8% and 79.3% were already released in SSF and SGF, respectively. The diffusion exponent *n* has values less than 0.43, indicating that the release of the phenolic compounds follows Fick’s diffusion law. In addition, the value of the diffusion exponent may indicate the form of the matrix from which the release of an active component is monitored. In this case, it indicates that the microbeads have a spherical shape [27].

In contrast to the other samples, the release of phenolic compounds from SA + GEL microbeads could be described exclusively by the Higuchi model (Figure 7d). This model is also based on Fick’s diffusion law and was developed to describe the release from different porous matrices, especially for the release of poorly soluble drugs encapsulated in semi-solid or solid matrices. Moreover, according to this model, the release of active ingredients is increased with decreasing tortuosity [113]. It is possible that due to the concentrations of GEL and SA used in the encapsulation process, strong ionic interactions of the polymers occur, resulting in increased tortuosity of the hydrogels. This could explain the lower release of total phenolic compounds in the first 123 min. Later, swelling of the dry microbeads occurs, which may reduce the aforementioned tortuosity and start a stronger release of the phenolic compounds.

## 4. Conclusions

Phenol-rich grape pomace extracts of the Cabernet Sauvignon variety were successfully encapsulated by ionic gelation with investigated coatings, and the encapsulation efficiency was strongly influenced by the type of coating. Moreover, it is possible to influence the rate and site of release of phenolic compounds in the gastrointestinal tract by the choice of coating, which may affect their potential bioaccessibility and absorption, on which their biological activity in the body depends. The application of mathematical models to describe the release process contributed to a better understanding of the release mechanism of phenolic compounds during simulated digestion in vitro. This work provides important information for the successful development of various formulations (dietary supplements, pharmaceuticals, functional foods, etc.) containing encapsulated phenolic compounds used in the pharmaceutical and food industries. Since this work showed that the shape and size parameters changed after freeze-drying, future research will focus on investigating different methods of drying microbeads to release phenolic compounds, and applying additional types and concentrations of coatings to obtain an even more complete picture of this complex digestion process. In addition, the results obtained should be further investigated with more complex digestive systems that more closely simulate the human digestion and include the application of digestive enzymes, a reactor with peristaltic movements, and human cell lines.

## Figures and Tables

**Table 1:** Measured shape parameters, their calculation equations, and the description of each parameter.

Shape Parameters	Calculation	Description
Circularity	$\frac{4\cdot \pi \cdot A}{P^{2}}$	a measure of the deviation of the particle from the sphere
Roundness	$\frac{4\cdot A}{\pi \cdot {Feret}_{MAX}^{2}}$	represents the curvature of the edges and corners of the particle
Solidity	$\frac{A}{Convex\;area}$	a measure of the compactness or smoothness of a particle

*A*—area (mm^2^); *P*—perimeter (mm); Feret diameters (MAX, MIN)—the distance between two parallel tangents to the particle at an arbitrary angle (mm); convex area—curve that is rounded outward of hydrogels or microbeads like the exterior.

**Table 2:** Mathematical models used for the modeling of the release of total phenolic compounds from encapsulated grape pomace extracts by ionic gelation.

Model	Equation/ Model Parameters	Hypothesis	References
First order	$F=100\cdot \left(1-e^{{-k}_{1}\cdot t}\right)$ *F*—the fraction (%) of drug released in time *t* *k*_1_—first-order release constant	describes the release kinetics of water-soluble substances in porous matrices and ionizing oil- or water-soluble substances from W/O/W emulsions the concentration of bioactive substance released is proportional to the concentration of remaining bioactive substance in the matrix and decreases as a function of time	[27,42,43,44,45,46]
Higuchi	$F=k_{H}\cdot t^{0.5}$ *F*—the fraction (%) of drug released in time *t* *k*_H_—the Higuchi release constant	describes the release kinetics of water-soluble and encapsulated poorly soluble substances encapsulated in solid or semi-rigid matrices applicable to systems of different structure and geometry	[27,28,42,47,48,49]
Hixson–Crowell	$F=100\cdot \left[\left(1-{\left(1-k_{HC}\cdot t\right)}^{3}\right)\right]$ *F*—the fraction (%) of drug released in time *t* *k*_HC_—the Hixson–Crowell release constant	applies to systems where dissolution occurs in parallel to the surface of the dosage form (e.g., tablets) surface area decreases proportionally with time and the geometric shape remains constant	[27,28,42,47,50]
Korsmeyer–Peppas	$F=k_{KP}\cdot t^{n}$ *F*—the fraction (%) of drug released in time *t* *k*_KP_—the release constant including geometric and characteristics of the bioactive dosage form *n*—the diffusional exponent (release dependent)	applicable when the release mechanism is unknown or when more than one release mechanism is involved designed for the release of a drug from a polymer matrix (e.g., hydrogel)	[27,42,51]

**Table 3:** Chemical composition of grape pomace Cabernet Sauvignon variety.

Component	Content (Mean ± SD%_db_)
Ash	4.89 ± 0.04
NDF	50.33 ± 1.69
ADF	40.03 ± 2.35
ADL (lignin)	25.80 ± 0.92
Hemicellulose	10.31 ± 0.96
Cellulose	14.22 ± 1.79
Crude proteins	7.38 ± 0.13
TOC_GP_	1.27 ± 0.01
TOC_LGPE_	35.63 ± 1.14
TN	1.27 ± 0.01
Free fats	8.18 ± 0.59
Glucose	4.53 ± 0.06
Arabinose	1.35 ± 0.46
Sucrose	3.23 ± 0.11
Fructose	8.51 ± 0.06
TPC	53.55 ± 2.81
TF	22.85 ± 0.12
TPA	9.79 ± 0.12

NDF—neutral detergent fibers, ADF—acid detergent fibers, ADL—acid detergent lignin, TOC_GP_—total organic carbon in grape pomace, TOC_LGPE_—total organic carbon in liquid grape pomace extract, TN—total nitrogen, TPC—total phenolic content, TF—total flavonoids content, TPA—total extractable proanthocyanidins, db—dry basis. All data are expressed as means value of replication (n = 3) ± SD (%_db_).

**Table 4:** Antioxidant activity of grape pomace extracts using DPPH and ABTS methods and its reducing power investigated with FRAP assay.

Method	Antioxidant Activity (mg_TROLOX_/g_db_)
DPPH	70.46 ± 0.24
FRAP	78.34 ± 1.55
ABTS	313.83 ± 41.89

Values are expressed as Trolox equivalent on dry basis (db) of grape pomace as mean values ± SD.

**Table 5:** Content of individual phenolic (C) in GP extract determined by UHPLC analysis.

	Compounds	C (μg/g_db_)
Phenolic acids (Hydroxybenzoic acids)	Gallic acid	383.41 ± 2.23
	3,4-Dihydroxybenzoic acid	90.34 ± 3.60
	*p*-Hydroxybenzoic acid	3.92 ± 0.00
	Syringic acid	76.35 ± 4.87
	Vanillic acid	17.73 ± 0.37
	Ellagic acid	127.34 ± 1.91
Phenolic acids (Hydroxycinnamic acids)	Caffeic acid	4.28 ± 0.21
	Ferulic acid	9.34 ± 0.24
	*o*-Coumaric acid	5.14 ± 0.03
	*p*-Coumaric acid	6.52 ± 0.15
Flavan-3-ols	Epicatechin	1279.11 ± 8.31
	Catechin	3088.84 ± 2.10
	Epicatechin gallate	71.27 ± 2.50
	Gallocatechin gallate	460.58 ± 3.00
	Procyanidin B1	843.44 ± 46.13
	Procyanidin B2	395.00 ± 16.90
Flavonols	Quercetin	470.69 ± 6.46
	Kaempferol	37.65 ± 1.08
	Rutin	12.89 ± 3.80
Stilbenes	Resveratrol	29.82 ± 0.66
	*ε*-Viniferin	62.27 ± 3.47
Anthocyanins	Oenin chloride	509.16 ± 3.35
	Myrtillin chloride	42.05 ± 0.40
	Kuromanin chloride	8.24 ± 0.15
	Petunidin chloride	8.39 ± 0.49
	Callistephin chloride	3.59 ± 0.21
	Peonidin-3-*O*-glucoside chloride	63.54 ± 0.61

All data are expressed as means value of replication (n = 3) ± SD.

**Table 6:** Values of parameters describing the size (area, perimeter, Feret), shape (circularity, roundness, solidity), and texture (hardness) of the hydrogel and freeze-dried microbeads containing phenol-rich grape pomace extract, prepared using sodium alginate (SA), SA and gelatin (SA + GEL), SA and gum arabic (SA + GA), and SA and chitosan (SA/CHIT) as coatings.

Sample	Size Parameters				Shape Parameters			Texture
	Area (mm^2^)	Perimeter (mm)	Feret_MAX_ (mm)	Feret_MIN_ (mm)	Circularity	Roundness	Solidity	Hardness (N)
Hydrogel microbeads								
SA	11.84 ± 0.77	13.21 ± 0.51	4.24 ± 0.19	3.65 ± 0.18	0.85 ± 0.04	0.89 ± 0.06	0.98 ± 0.00	0.40 ± 0.07
SA + GEL	16.56 ± 1.06	16.09 ± 1.15	4.99 ± 0.39	4.39 ± 0.16	0.81 ± 0.07	0.90 ± 0.06	0.97 ± 0.02	0.39 ± 0.10
SA + GA	14.61 ± 0.90	14.50 ± 0.46	4.62 ± 0.15	4.11 ± 0.19	0.87 ± 0.02	0.91 ± 0.05	0.98 ± 0.01	0.32 ± 0.03
SA/CHIT	14.66 ± 0.87	14.61 ± 0.55	4.63 ± 0.18	4.13 ± 0.22	0.86 ± 0.03	0.92 ± 0.05	0.98 ± 0.01	0.29 ± 0.07
Freeze-dried microbeads								
SA	4.50 ± 1.15	8.57 ± 0.84	2.84 ± 0.26	2.17 ± 0.30	0.76 ± 0.07	0.79 ± 0.09	0.94 ± 0.03	3.40 ± 3.52
SA + GEL	10.26 ± 0.80	13.45 ± 0.72	4.11 ± 0.33	3.38 ± 0.19	0.72 ± 0.06	0.85 ± 0.08	0.96 ± 0.01	0.98 ± 0.66
SA + GA	10.12 ± 0.78	13.46 ± 1.08	4.12 ± 0.28	3.34 ± 0.22	0.71 ± 0.08	0.83 ± 0.09	0.96 ± 0.01	1.22 ± 0.43
SA/CHIT	8.16 ± 1.47	11.63 ± 1.14	3.70 ± 0.33	2.99 ± 0.29	0.76 ± 0.06	0.83 ± 0.07	0.96 ± 0.01	0.38 ± 0.10

**Table 7:** Estimated parameters of the applied mathematical models for describing the release kinetics of phenolic compounds (*k*_1_, *k*_H_, *k*_HC_, *k*_KP_—release constants for the corresponding model; *n*—the diffusional exponent) from freeze-dried microbeads containing grape pomace extract prepared with different coatings, and statistical criteria for the success of the model approximation (R^2^adj—the adjusted coefficient of determination, AIC—the Akaike information criterion, MSC—the model selection criterion).

Mathematical Model	Release Rate Constants and Statistical Criteria of Model Approximation Success				
		SA	SA/CHIT	SA + GA	SA + GEL
First-order model	R^2^_adj_	0.692	0.831	0.690	0.815
	AIC	133.945	109.170	114.796	108.980
	MSC	0.548	1.155	0.590	1.313
	*k_1_*	0.029	0.028	0.018	0.009
Higuchi model	R^2^_adj_	0.680	0.763	0.836	0.895
	AIC	134.613	113.980	105.891	101.097
	MSC	0.506	0.811	1.226	1.876
	*k_H_*	7.905	7.742	6.874	5.819
Hixson–Crowell model	R^2^_adj_	0.538	0.721	0.609	0.765
	AIC	140.504	116.260	118,069	112.351
	MSC	0.138	0.649	0.356	1.072
	*k_HC_*	0.006	0.006	0.004	0.003
Korsmeyer–Peppas model	*R* ^2^_adj_	0.973	0.973	0.966	0.808
	AIC	95.693	84.141	84.535	110.410
	MSC	2.939	2.943	2.751	1.211
	*k_KP_*	28.240	22.548	20.290	11.670
	*n*	0.236	0.288	0.275	0.338

SA—sodium alginate; SA/CHIT—sodium alginate and chitosan; SA + GA—sodium alginate and gum arabic; SA + GEL—sodium alginate and gelatin. The green marked parameters indicate a model that best fits the experimental data.

## Data Availability

Not applicable.

## Supplementary Materials

The following supporting information can be downloaded at: https://www.mdpi.com/article/10.3390/pharmaceutics15030980/s1, Figure S1: Kinetics of phenolic compound release from freeze-dried microbeads containing grape pomace extracts prepared with (a) sodium alginate (SA); (b) sodium alginate with chitosan (SA/CHIT); (c) sodium alginate with gum arabic (SA + GA); and (d) sodium alginate with gelatin (SA + GEL) (symbols—experimental data, lines—approximate curves according to different mathematical models).
