# Supplementary material for: Physicochemical Characterization and Evaluation of Gastrointestinal In Vitro Behavior of Alginate-Based Microbeads with Encapsulated Grape Pomace Extracts
Source: Pharmaceutics. 2023 Mar 18;15(3):980. doi: 10.3390/pharmaceutics15030980 (PMC10052734; doi:10.3390/pharmaceutics15030980)
Supplement: Supplementary file 1 [file pharmaceutics-15-00980-s001.zip › pharmaceutics-2135262-supplementary.pdf]

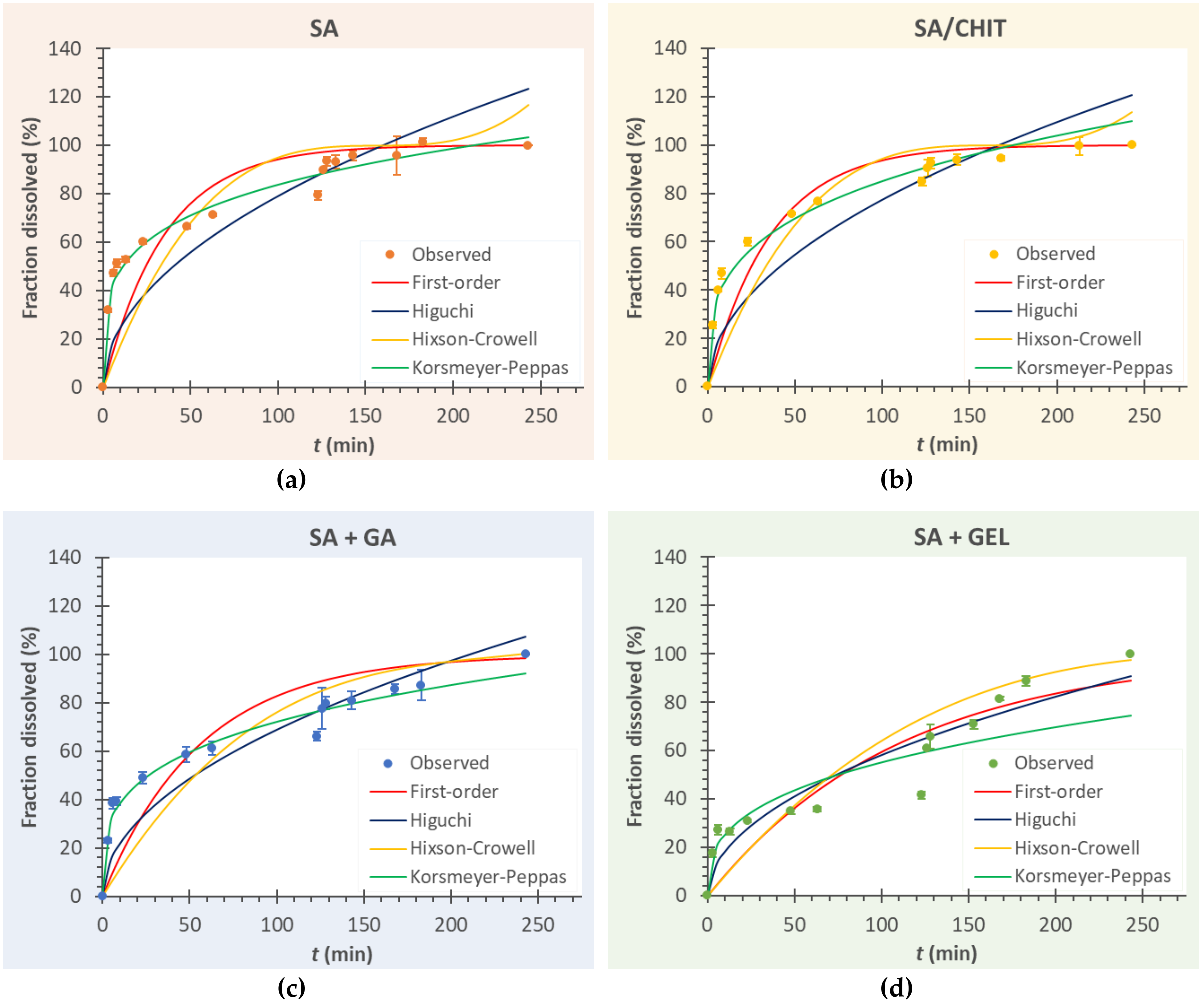

Figure S1. Kinetics of phenolic compound release from freeze-dried microbeads containing grape pomace extracts prepared with (**a**) sodium alginate (SA); (**b**) sodium alginate with chitosan (SA/CHIT); (**c**) sodium alginate with gum arabic (SA + GA); and (**d**) sodium alginate with gelatin (SA + GEL) (symbols - experimental data, lines - approximate curves according to different mathematical models).
